# Automated segmentation of liver segment on portal venous phase MR images using a 3D convolutional neural network

**DOI:** 10.1186/s13244-022-01163-1

**Published:** 2022-02-24

**Authors:** Xinjun Han, Xinru Wu, Shuhui Wang, Lixue Xu, Hui Xu, Dandan Zheng, Niange Yu, Yanjie Hong, Zhixuan Yu, Dawei Yang, Zhenghan Yang

**Affiliations:** 1 Department of Radiology, Beijing Friendship Hospital, Capital Medical University, Beijing, China; 2 Weihai Municipal Hospital, Cheeloo College of Medicine, Shandong University, Weihai, China; 3 Shukun (Beijing) Technology Co., Ltd., Beijing, China

**Keywords:** Liver segment segmentation, 3D-CNN, Couinaud classification, MRI

## Abstract

**Objective:**

We aim to develop and validate a three-dimensional convolutional neural network (3D-CNN) model for automatic liver segment segmentation on MRI images.

**Methods:**

This retrospective study evaluated an automated method using a deep neural network that was trained, validated, and tested with 367, 157, and 158 portal venous phase MR images, respectively. The Dice similarity coefficient (DSC), mean surface distance (MSD), Hausdorff distance (HD), and volume ratio (RV) were used to quantitatively measure the accuracy of segmentation. The time consumed for model and manual segmentation was also compared. In addition, the model was applied to 100 consecutive cases from real clinical scenario for a qualitative evaluation and indirect evaluation.

**Results:**

In quantitative evaluation, the model achieved high accuracy for DSC, MSD, HD and RV (0.920, 3.34, 3.61 and 1.01, respectively). Compared to manual segmentation, the automated method reduced the segmentation time from 26 min to 8 s. In qualitative evaluation, the segmentation quality was rated as good in 79% of the cases, moderate in 15% and poor in 6%. In indirect evaluation, 93.4% (99/106) of lesions could be assigned to the correct segment by only referring to the results from automated segmentation.

**Conclusion:**

The proposed model may serve as an effective tool for automated anatomical region annotation of the liver on MRI images.

## Key points

- 3D-CNN exhibits satisfactory performance for segmentation of liver-segment on MR images.
- It is robust across different MR scanners and variable liver backgrounds.
- It lays a foundation for computer-aided analyses of liver MR images.

## Introduction

Subdivision of the human liver into anatomical regions is part of the daily routine of radiologists, especially considering the need for precise preoperative localization of focal liver lesions. Currently, the type of hepatectomy chosen depends mainly on the segmental localization of the lesion [1]. In addition, the identification of liver segment is also crucial to reduce the risk of liver surgery [2]. Several quantitative parameters, such as liver fat fraction and R2* value, also need to be measured at a segment level due to the heterogeneous nature of some diseases in liver [3]. These tasks are usually performed by radiologists via visual interpretation or manual segmentation, which are labor-intensive, time-consuming, and prone to intra- and inter-observer variations. A practicable automated liver segment segmentation tool is therefore very desirable for clinical purposes.

In the past two decades, a lot of research work has been done in computer-assisted liver segment segmentation [4–21], most of them using traditional machine learning techniques [4, 7–21], which cannot meet the needs of clinical applications in terms of segmentation performance and efficiency. The method proposed by Lebre et al. [8] requires first segmentation of the hepatic vessels using the skeletonization process, and then the main direction of the largest vessels was extracted to achieve separation of different liver segments. However, the whole process took more than 8 min, and good results are highly dependent on accurate vascular segmentation, which is difficult owing to the complex intertwined vascular anatomy in the liver. To tackle this problem, in a recently published paper, Wu et al. [4] introduced a multiple feature-based method. Their method improved the accuracy of vessel separation and was subsequently expected to improve the segmentation of liver segments. However, the quantitative results of liver segments segmentation were not given in this paper and their method took an average of 20.8 s per case to obtain liver segments.

In recent years, the rapid development of deep learning, particularly the advent of convolutional neural network (CNNs), has pushed medical image segmentation to a new level [22]. Tian et al. [5] used a 2.5-dimensional (2.5D) class-aware deep neural network with spatial adaptation to obtain Couinaud segmentation of the liver from CT images. They achieved an average dice score of 0.882 and the entire running time was quite fast (~5 s). The results demonstrated the potential of deep learning to improve segmentation efficiency and accuracy over traditional methods. For MR images, Mojtahed et al. [6] recently reported a Couinaud segment volume measurement tool based on deep learning, but this device is semi-automatic (needs human position eight landmark placement). To our knowledge, there is still lack of a deep learning-based fully automatic tool for liver segments segmentation on MR images. In this study, we would like to develop a 3D U-net based algorithm to automatically segment the liver into Couinaud regions on MR images.

## Materials and methods

### Dataset

This retrospective study was approved by the institutional review board of Beijing Friendship Hospital, and informed consent was waived. All abdominal MR scans were performed as part of routine clinical care for patients. For model development, we initially obtained 744 multiphase contrast-enhanced MR scans by searching picture archiving and communication system (PACS) randomly between January 2017 and February 2019. After excluding the following cases: (1) Those with apparent liver deformation due to advanced cirrhosis or large tumors (*n* = 33). (2) Those with poor imaging quality or severe artifacts (n=8), and (3) those after partial hepatectomy or liver transplantation (*n* = 21). A total of 682 MR scans were included in the final cohort (367, 157, 158 for training, validation and testing, respectively).

For clinical evaluation, a consecutive sample of 100 patients who underwent abdominal enhanced MR in our hospital between April 1 and 24, 2021 was collected.

### Image acquisition

One 1.5T and four 3.0T MR scanners (GE, GE Health care, Boston, USA; Siemens Medical Solutions, Forchheim, Germany, Siemens Medical Solutions, Forchheim, Germany; Philips Medical Systems, Best, the Netherlands) were used in this study. 3D T1-weighted breath-hold sequences from our institutional standardized liver MR imaging protocols were used for contrast-enhanced imaging. Parameters varied across equipment and ranged from TR 3.4–4.1ms, TE 1.15–1.91ms, flip angle 10–15°, bandwidth 125 and 500 Hz, slice thickness 3–4.4 mm, image matrix 216 × 188 to 288 × 216 and field-of-view 380 × 342 mm to 400 × 369 mm, acquisition time 10 s. In this study, only portal venous phase (PVP) images acquired at ~60s postinjection were used. Anonymized MR images in DICOM format were exported from PACS.

### Manual segmentation

**Fig. 1:**
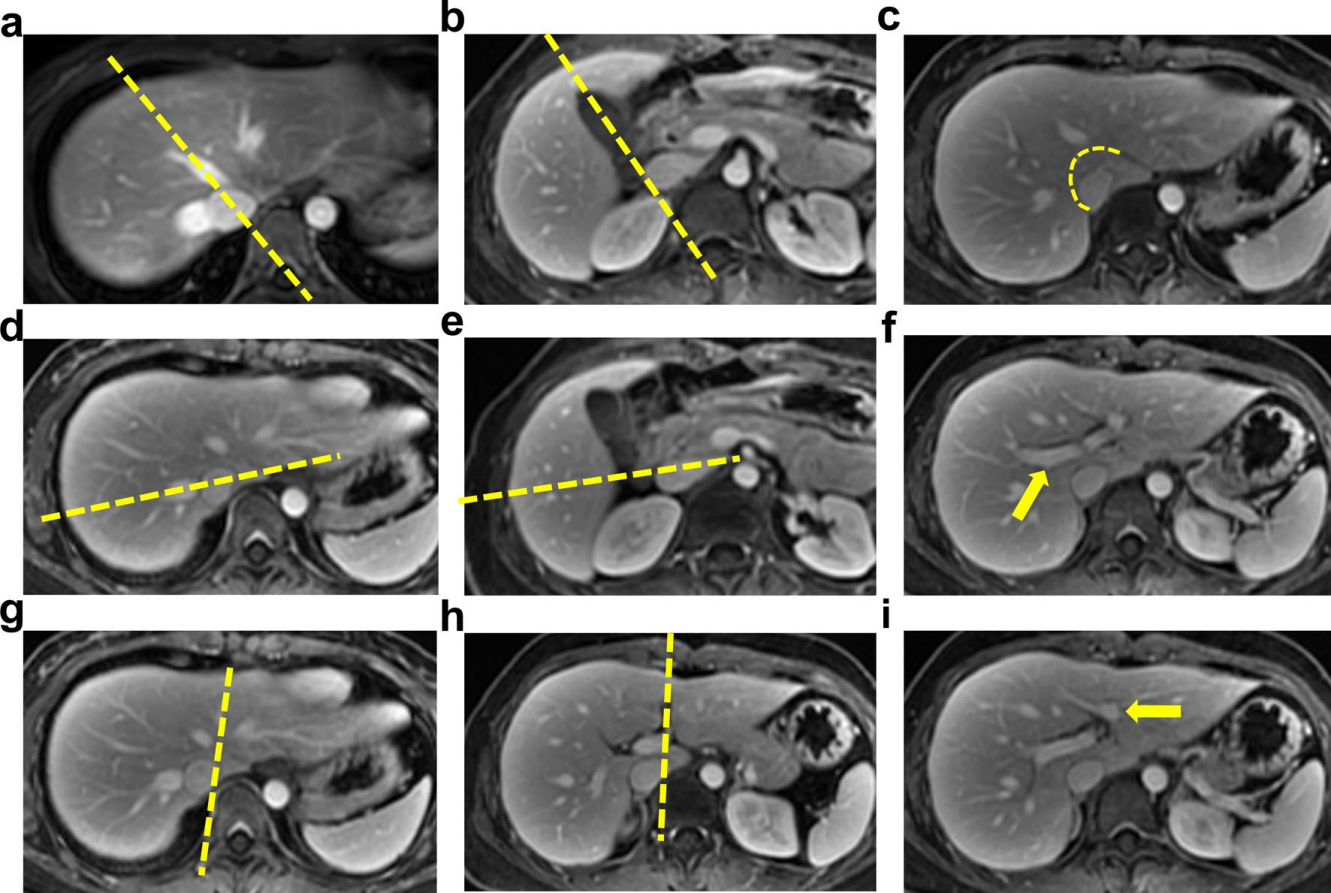
Method of Couinaud’s classification on axial MRI images. Five planes (three vertical planes along the main stem of hepatic veins and two horizontal planes through the primary branches of the portal vein) are used to divide the liver. In axial MR images, the line from the midpoint of the IVC to the MHV or vertex of the gallbladder fossa divides the right and left lobes (**a** and **b**). The RHV further divides the right lobe into anterior and posterior segments (**d** and **e**). The LHV (in the upper part of the liver) and the left longitudinal fissure (in the lower part of the liver) divide the left lobe into a medial and a lateral segment. (**g** and **h**). Each segment of right and left lateral lobes is further divided into a superior segment and an inferior segment by the main stem of the right portal vein and the sagittal part of the left portal vein (**f** and **i**). The caudate lobe is separated from the other segments by the natural curve from the venous ligament fissure to the right wall of the IVC (**c**). IVC, inferior vena cava; MHV, middle hepatic vein; RHV, right hepatic vein; LHV, left hepatic vein.

Classification of liver segment was based on Couinaud's description [23], as shown in Fig. 1. The ground-truth was generated by two radiologists (Z.Y.S. and Z.X.L.) with more than 5 years of experience in abdominal imaging using Mimics software (version 19.0; Materialise, Leuven, Belgium). All the images were divided into two groups and evenly distributed to each radiologist. To ensure the consistency of the segmentation results, two radiologists went through a training session and segmented several cases together before formal segmentation.

### Model development

We used 3D U-Net as the backbone, which is built with 3D conv, ReLU, BN and pooling layers [24]. The network takes a 3D image as input, and produces the output with the same shape as the input image. The images were normalized by spacing and cropped into a fixed shape (160 × 160 × 160) in a sliding window approach before feeding into the model. As the variation in the gray distributions of MR scan images should not be ignored compared to CT scans, a grayscale normalization by subtracting the mean and divide by the standard deviation was applied after spacing normalization.

**Fig. 2:**
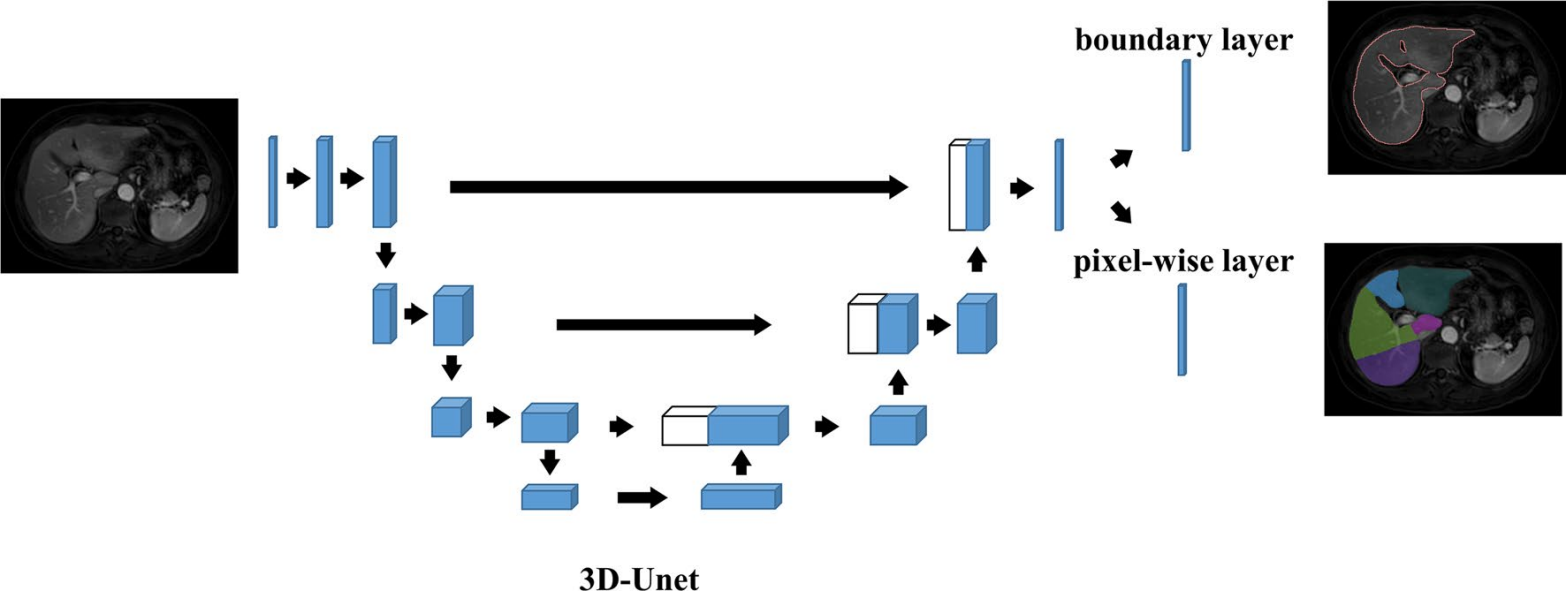
Network structure of the liver segment segmentation model.

We introduced two branches in the output layer. As shown in Fig. 2, the upper segment produces the liver boundary and the other segment is the pixelwise segmentation of the liver segments. The late output serves as the final output, while the previous output helps to produce a better boundary. The model is trained end-to-end using the SGD optimizer on the training dataset. The learning rate of the training is start at 1e−3 and decay by 0.1 at every 50 epochs. We use Dice-Loss as the loss function, and batch size is set to 8. The training is end at 150 epochs.

### Evaluation

#### Quantitative evaluation

Quantitative results were derived on the internal test dataset (158 cases not used for training). The following four parameters were used:

##### The Dice similarity coefficient (DSC)

DSC is used to measure the voxel overlap between the prediction (*X*) and the ground truth (*Y*). The value of this metric ranges from 0 to 1, with minimum and maximum values indicating no overlap and fully overlap, respectively.

$${\text{DSC}} = \frac{2|X \cap Y|}{{|X| + |Y|}} \times 100{{\% }}$$

##### The mean surface distance (MSD) and Hausdorff distance (HD)

MSD and HD are designed to quantify surface-based difference between* X* and* Y*. MSD calculates the mean distance between the two surfaces, whereas HD measures the maximum distance between them.

$$MSD\left( {X,Y} \right) = \frac{1}{{N_{X} + N_{Y} }}\left\{ {\mathop \sum \limits_{{x \in S_{X} }} \mathop {\min }\limits_{{y \in S_{Y} }} \left( {dist\left( {x, y} \right)} \right) + \mathop \sum \limits_{{y \in S_{Y} }} \mathop {\min }\limits_{{x \in S_{X} }} \left( {dist\left( {x, y} \right)} \right)} \right\}$$

$$HD\left( {X,Y} \right) = \max \left( {hd\left( {X,Y} \right),\;hd\left( {Y,X} \right)} \right)$$

$$hd \, \left( {X,Y} \right) = \mathop {\max }\limits_{x \in X } \mathop {\min }\limits_{y \in Y} \parallel x - y\parallel^{2}$$

$$hd \, \left( {Y, \, X} \right) = \mathop {\max }\limits_{y \in Y } \mathop {\min }\limits_{x \in X} \parallel x - y\parallel^{2}$$

##### The volume ratio (RV)

RV computes the volume ratio of the liver segments from two segmentations, defined as *RV* (*X*, *Y*) = *V*_*X*_/*V*_*Y*_, where *V*_*X*_ and *V*_*Y*_ represent the volume of model and manual segmentations, respectively.

The value of DSC, MSD, HD and RV for each liver segment and the average of eight segments were calculate. All of these quantitative parameters by different MRI manufacturers were also calculated. In addition, time consumed for AI-based segmentation and manual segmentation was compared.

#### Clinical evaluation

Our model was applied to a total of 100 consecutive patients who received upper abdominal contrast-enhanced MR examinations in our hospital from April 1 to 24, 2021. The clinical evaluation consists of the following two parts:

##### Qualitative evaluation

The segmentations generated by the model were evaluated independently by two radiologists (X.H. and Y.D.W.) with more than 15 years’ experience. Segmentation quality of each patient was classified as either good, moderate, or poor based on the following two main elements: a. The accuracy of separation planes placement; b. whether inter-segment segmentation fault occurs. Accuracy of separation planes placement was determined by how much they shifted off center. For the three vertical planes along the hepatic veins, a shift ≤ 5 mm was defined as slight, 5–10 mm as moderate and ≥ 10 mm as severe. For the two horizontal planes along the portal veins, a shift ≤ 2 slices was defined as slight, 2–5 slices as moderate and ≥ 5 slices as severe. Inter-segment segmentation faults are the errors that cannot be explained by misplacement of the separation planes and are usually irregular shaped. Finally, segmentations with two or less slight shifts and no other errors, or with only one moderate shift and no other errors were classified as good; Those with more than two slight shifts or more than one moderate shifts, but no severe plane shifts and no inter-segment segmentation faults, were classified as moderate. Once the segmentation had a severe shift or inter-segment segmentation fault, it was classified as poor segmentation. Differences were rerated by an abdominal subspecialty radiologist (Y.Z.H.) with more than 30 years of experience.

##### Indirect evaluation

**Fig. 3:**
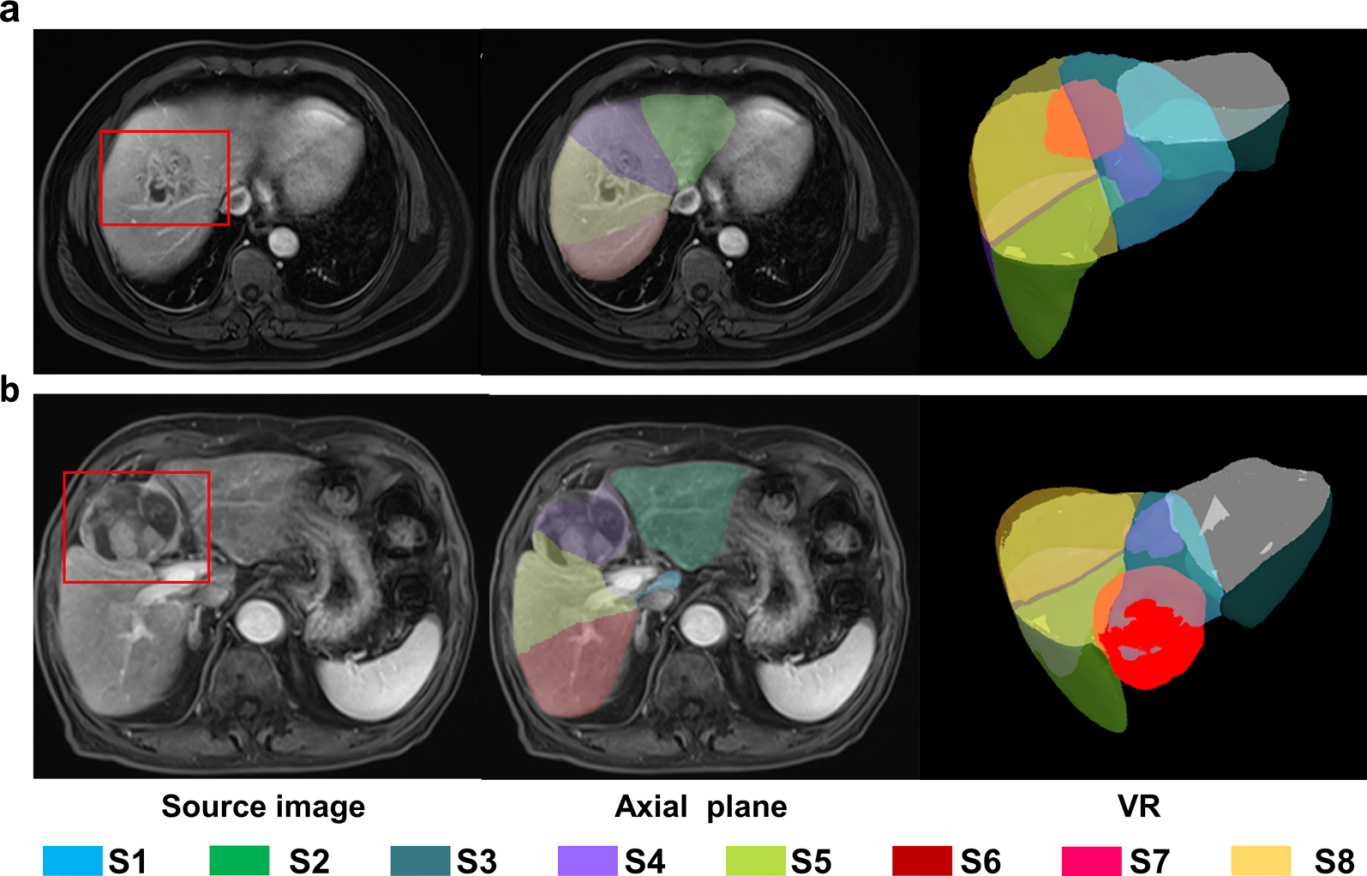
Examples of indirect evaluation. **a** A liver abscess that resided in S4 and S8 was assigned to the correct segments by AI. **b** Shows a large tumor, located in S4 with slight pushing of the margin of S8. However, if we only refer to the AI segmentation result, it will be assigned to S4 and 8. The red boxes in the source image and bright red volumes in VR images indicate the lesions. AI, artificial intelligence; S, segment; VR, volume rendering.

The accuracy of segmentation was assessed indirectly based on the localization of lesions in the liver. The steps were as follows: first, cases with uncountable lesions or no lesions were excluded. Then, for each of the remaining cases, all the focal abnormal findings (larger than 5 mm) that could be seen in PVP were identified. If a liver had more than five lesions, only the five largest lesions were selected. Finally, for each eligible lesion, the specific liver segment that it resides (by only referring to the automated segmentation results) was recorded and judged by radiologists. Detection, screening, and localization judgment of lesions were performed by two radiologists (X.H. and Y.D.W.) in consensus. One radiologist (Y.Z.H.) arbitrated in case of disagreement. Fig. 3 illustrates the indirect evaluation with two examples.

### Statistical analysis

Continuous variables were expressed as mean ± standard deviation (SD) or as the median and interquartile range depending on the normality of the data. Categorical variables were summarized as frequencies and percentages. Reader agreement was tested by the linearly weighted kappa coefficient. All statistical analyses were conducted using SPSS software version 25.0 (IBM, Armonk, New York). Significance was set at *p *< 0.05.

## Results

### Patients and image characteristics

A total of 782 patients who underwent multiphase contrast-enhanced MR scans at our hospitals were included in this study. The mean age was 59 ± 15 years, and there were slightly more males (404; 51.7%). The demographic and MR scan profiles that were employed for each dataset are summarized in Table 1.

**Table 1:** Basic characteristics of the enrolled subjects for model development and clinical evaluation set.

Parameters	Model development			Clinical evaluation
	Training	Validation	Test	
Number of patients	367	157	158	100
Male/female	189/178	81/76	83/75	51/49
Age (year)	60 ± 16	58 ± 15	60 ± 14	59 ± 13
Different manufactures (GE/Siemens/Philips)	203/107/57	94/32/31	105/28/25	48/52/0

### Quantitative results

Experimental results show that our method is capable of accurately subdividing the input liver MR images into eight anatomical regions, with an average DSC of 90.20%. The good value of other metrics including HSD, MSD, and RV further confirmed the accuracy of the proposed method. Tables 2 and 3 summarize all four metrics (DSC, MSD, HD, and RV) on the whole dataset and on the data by different MR manufacturers, respectively. As shown in Table 3, there were no significantly difference of the quantitative evaluation results determined by this segmentation model on the data from different manufacturers.

**Table 2:** Quantitative evaluation for automated liver segment segmentation.

Metrics	S1	S2	S3	S4	S5	S6	S7	S8	AVG
DSC (%)	93.15	92.24	91.03	92.18	88.07	87.53	89.28	88.11	90.20
MSD (mm)	1.21	3.31	2.98	2.44	4.21	4.52	3.99	4.03	3.34
HD (mm)	1.39	3.82	3.25	2.89	4.33	4.67	4.21	4.32	3.61
RV	1.08	1.06	0.96	0.93	1.18	0.89	0.91	1.09	1.01

*S* segment, *AVG* average, *DSC* dice similarity coefficient, *MSD* mean surface distance, *HD* Hausdorff distance, *RV* volume ratio.

**Table 3:** Quantitative evaluation results by different MR manufacturers.

Metrics	S1	S2	S3	S4	S5	S6	S7	S8	AVG
GE									
DSC (%)	93.33	92.01	90.93	92.43	89.02	87.36	88.97	88.42	90.31
MSD (mm)	1.18	3.33	3.01	2.38	3.71	4.62	4.11	3.93	3.28
HD (mm)	1.34	3.86	3.33	2.79	4.02	4.76	4.32	4.22	3.58
RV	1.02	1.04	1.02	0.98	1.11	0.93	0.95	0.95	1.00
Siemens									
DSC (%)	93.25	92.11	91.20	92.24	88.33	87.50	89.06	88.41	90.26
MSD (mm)	1.17	3.29	2.97	2.38	4.17	4.53	4.10	3.97	3.32
HD (mm)	1.35	3.81	3.20	2.87	4.28	4.69	4.42	4.22	3.61
RV	1.05	1.07	0.97	0.92	1.14	0.90	0.90	1.05	1.00
Philips									
DSC (%)	92.28	93.35	91.26	91.06	83.79	88.28	90.83	86.47	89.67
MSD (mm)	1.38	3.24	2.86	2.75	6.35	4.08	3.36	4.51	3.61
HD (mm)	1.64	3.66	2.96	3.33	5.68	4.26	3.51	4.85	3.73
RV	1.36	1.13	0.69	0.73	1.51	0.71	0.75	1.72	1.06

*S* segment, *AVG* average, *DSC* dice similarity coefficient, *HD* hausdorff distance, *MSD* mean surface distance, *RV* volume ratio.

The average running time using the developed model on an GPU-accelerated computing platform with Xeon(R) Silver 4210R (CPU) and GeForce RTX 2070 (GPU) was 8.2 ± 2.4 s. This takes much less time than manual segmentation, which took 26 min on average for a set of liver MR images.

## Clinical evaluation results

### Qualitative evaluation

**Fig. 4:**
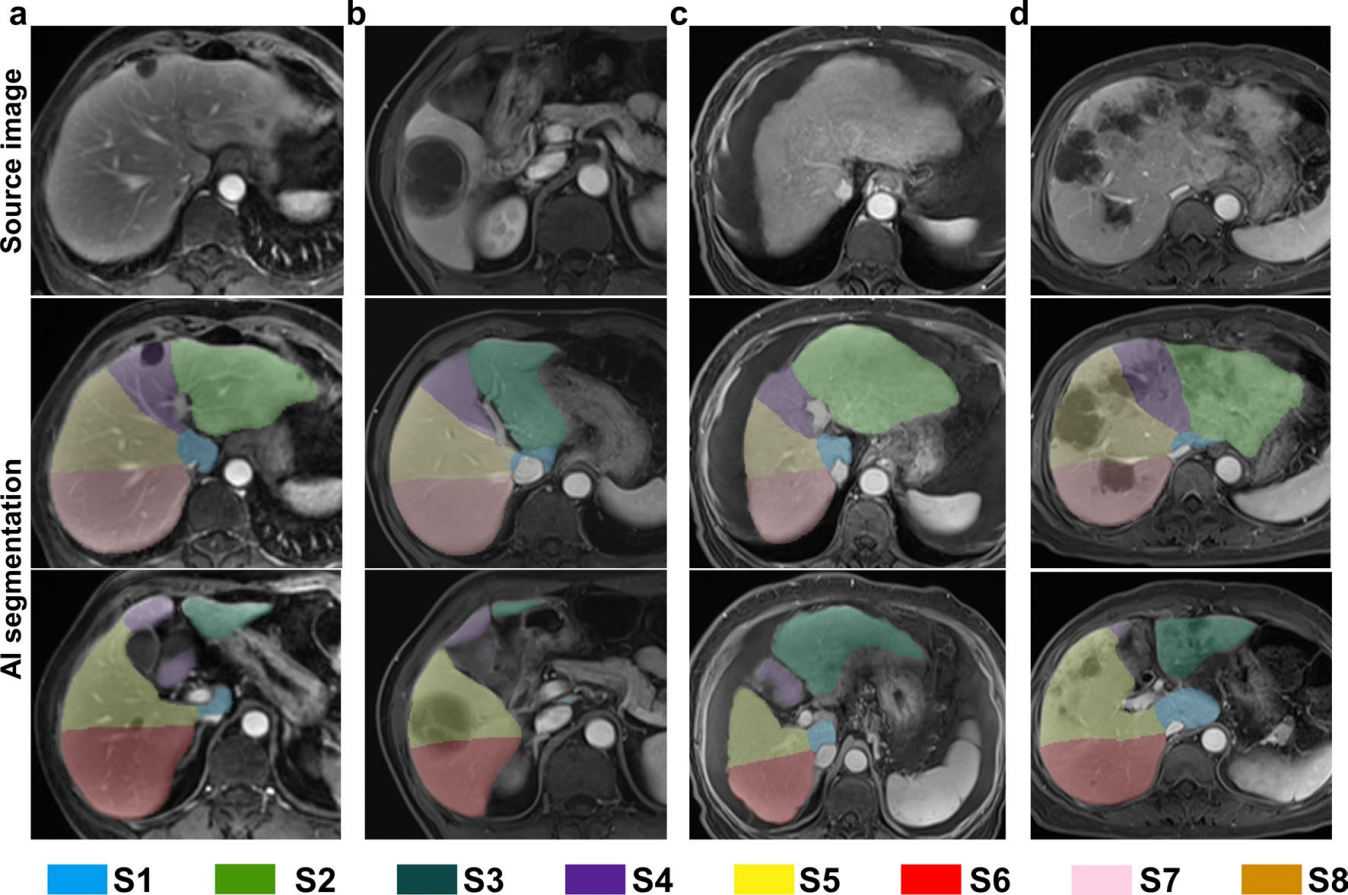
Examples of successful segmentations from AI in the clinical evaluation set. **a** A good liver background with several hepatic cysts in separate segments. **b** A case of a large solitary liver abscess occupying 2 segments of the liver (S5 and S6). **c** Liver cirrhosis. **d** Liver metastases (multiple masses and nodules in the liver). The top row shows the original images. The middle row shows the upper part of the liver with segmentation masks from AI, and the last row shows the lower part of the liver. AI, artificial intelligence; S, segment.

**Fig. 5:**
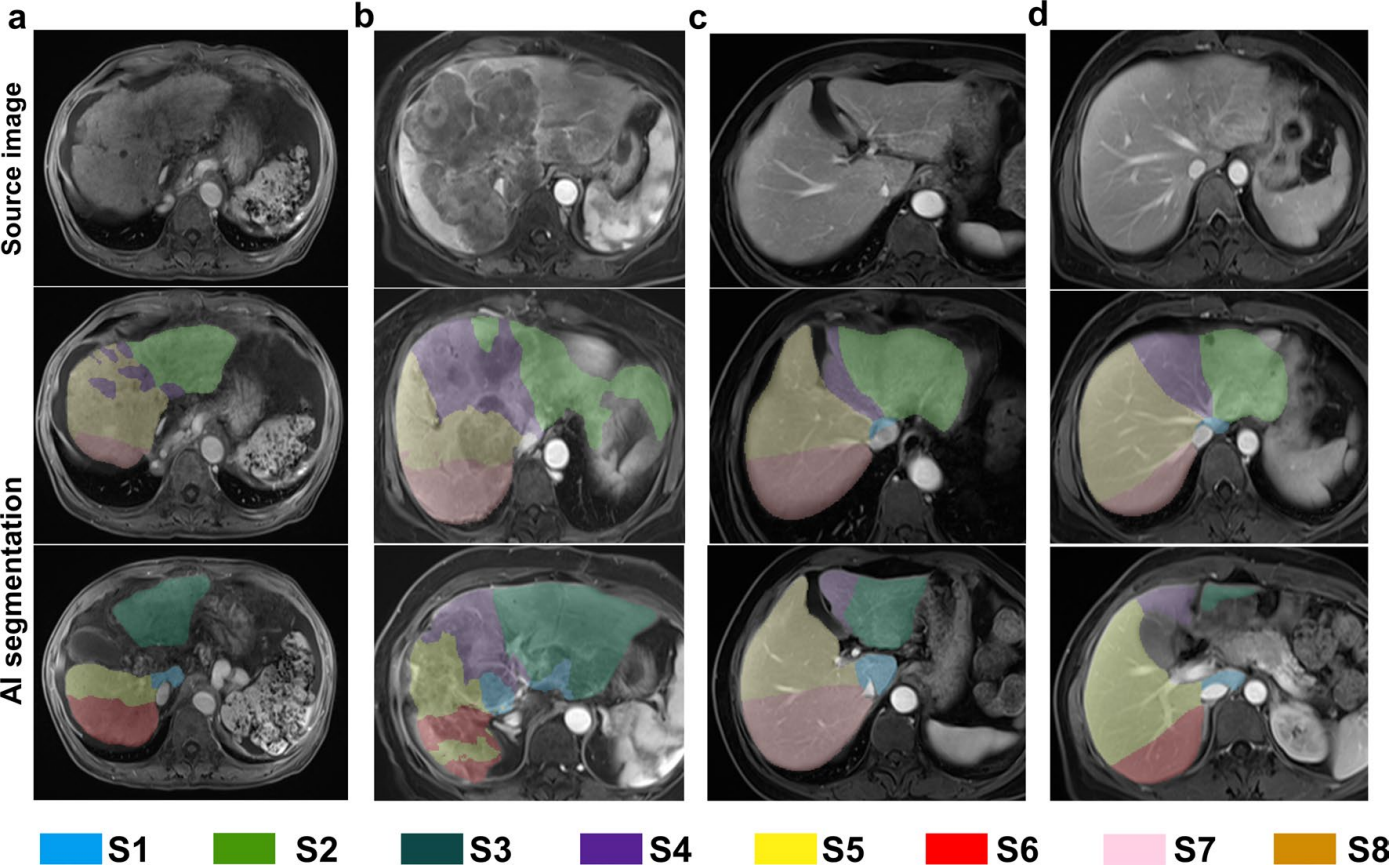
Examples of erroneous segmentations from AI in the clinical evaluation set. **a** A case of liver cirrhosis. Obvious intersegment misidentification occurred in the liver right lobe. Morphological changes in the liver and poor visualization of the hepatic veins can be observed in the corresponding source image. **b** A case of liver metastases. Oversegmentation errors occurred in the whole liver. Intrahepatic vasculature was diffusely invaded by metastases. **c** A case of gallbladder in ectopic locations. The gallbladder lies in the hepatic longitudinal fissure instead of the gallbladder fossa. Most likely for this reason, S4 was assigned to the left lobe by AI segmentation. **d** A case with hepatic vein variation. AI might mistake the large right posterior hepatic marginal vein for the right hepatic vein, which causes a rightward shift of the border line between the right anterior lobe and right posterior lobe. AI, artificial intelligence; S, segment.

The concordance between the two raters was satisfactory (kappa = 0.858). 6% (6/100) of the cases were rated as poor, meaning they had more serious segmentation errors that needed to be manually corrected. Among the six cases with poor segmentation, five cases were found for the patients with cirrhosis, and 1 case had diffuse metastases in the liver. Severe changes in liver shape and vascular anomalies caused by liver diseases appeared to be the main reason for the poor segmentation. Some examples of AI segmentations from different liver conditions are presented in Figs. 4 and 5.

### Indirect evaluation

After excluding 46 cases with no or uncountable lesions, a total of 106 lesions from 54 patients were used to evaluate the accuracy of AI segmentation. The maximal diameters of all lesions ranged from 0.6 to 6.0 cm (mean +/− SD, 1.6 cm +/− 0.8 cm) on MRI. According to the results, 93.4% (99/106) of the lesions were assigned to the segments where they were actually located. Moreover, the proposed method not only accurately located small lesions in a single segment but was also effective for massive lesions across segments. Seven of 106 lesions in total failed to be accurately located, of which two were due to the severe segmentation errors. Of the other errors, four lesions were on the boundary between two segments, and one lesion was on the edge of the liver and was not included in the segmentation.

## Discussion

In this study, we proposed and validated a deep learning model for fully-automated Couinaud segmentation of the liver based on PVP MRI images. Current experimental results reveal that our model can accurately subdivide the input liver MR images into eight anatomical regions and is robust across different MR scanners. The effectiveness of the proposed method was also validated in clinical scenarios and it would be a promising tool for assisting lesion localization automatically.

Subdivision of the liver into anatomical regions is critical because it is a fundamental step in potential computer-aided diagnosis in liver imaging. Most researchers who work on liver segment segmentation present their results on CT. Although CT is more widely used, MRI has several very definite advantages and can be used as a crucial complement to CT to screen for and diagnose liver disease [25, 26]. Lebre et al. [8] introduced a method for liver segment segmentation that is applicable to MR images. However, it requires liver segmentation and extraction of the vascular network before the Couinaud classification can finally be obtained. The time cost of the whole process is more than 8 min. Furthermore, their model performance was inadequately validated on a very small dataset (4 cases) and no quantitative results were given. Zhang et al. [9] proposed a method for partitioning the liver on 4D dynamic contrast-enhanced MR (DCE-MR) imaging. They obtained a mean DSC value of 88.43%. The accuracy is acceptable considering that it is produced on DCE-MR images with low spatial resolution. However, their method is not fully automated as manual feature extraction is needed in the process. In this work, our fully-automated approach received a quantitative assessment on a larger testing dataset, and the results confirmed that it is efficient, accurate and robust on three heavyweight MR manufacturers. We believe this is a step forward toward achieving intelligent diagnosis for liver MR images.

Previously, the evaluation of liver segment segmentation was mostly performed by visual inspection [7] and volumetric validation [16–18, 20, 21], or a combination of both [10, 19]. It is a major challenge because it imposes to have actual segment specimens. Several recent studies have adopted new approaches, including quantitative assessment, qualitative assessment, and indirect assessment [4, 5, 8, 9]. However, they all have a small sample size problem. In this study, we made a thorough evaluation of the model by using three different methods. Quantitative evaluation provides exact results and allows comparison between different studies. A common metric used for this purpose is DSC. To date, the accuracy of liver segment segmentation produced by prior methods does not exceed a DSC of 88.2% [5]. In this work, our model yielded a mean DSC of 90.2%. Good results were also obtained in HD, MSD and RV, metrics that have not yet been reported in similar research but have been widely used for segmentation evaluation. As a part of our experiment, the model was applied to real clinical scenarios for evaluation. As expected, our model generated good segmentation images in most cases, covering a wide range of liver appearances from normal to abnormal. It is worth mentioning that some cases with advanced liver cirrhosis or multiple liver metastases were also accurately segmented by our model and some of the large across-segment tumors did not affect the detection of boundary lines. Poor segmentation occurred mainly in cases with severe vascular problems, including abnormal vascular routes or vessels that were badly invaded by tumors. This problem may be solved by collecting this kind of case and retraining the model. Additionally, our approach has shown promising results in assisting lesion localization. As shown, 93.4% of the lesions were localized in the right segments simply by referring to the segmentation results without using any medical experience. This is meaningful considering the important role of automated lesion localization for future liver computer-aided diagnosis systems. Further investigating the mislocalized lesions, we found that most of them were at the edge of segment and pushed the borderline between two adjacent segments (Fig.3b). They were often mistaken for a cross-segmental lesion according to automated segmentation results. Only a few mislocalizations were caused by the real severe segmentation errors of liver segments.

Although the results are promising, our study still have several limitations. First, this is a retrospective single-institution study. Although the evaluation dataset maximally simulated real clinical scenario, more image data that collected prospectively from multiple centers are still necessary to assess the model performance. Second, manual segmentation of the qualitative evaluation dataset was produced by radiology residents rather than experienced specialists. Nonetheless, it has certain rationality because residents are often the ones who do segmentation work in clinical practice. Third, the performance of the model segmentation was validated only on PVP MR images. Future work should include extending the method to more sequences and modalities. Fourth, although the acceptable level of liver deformities by our method is good, it is still limited in cases in which the liver vasculature is severely deformed or has poor visibility. We will conduct future work on this problem.

In conclusion, we designed a 3D U-Net model by jointly adopting the pixelwise losses and boundary loss for liver segment segmentation on MRI images. A thorough evaluation from three respects was conducted, showing promising segmentation performance. We anticipate that this practical tool will assist in planning surgical strategies and enhancing computer-aided quantitative analyses of liver MR images in the future.

## Data Availability

The datasets used and/or analyzed during the current study are available from the corresponding author on reasonable request.

## Abbreviations

AVG: Average
CNN: Convolutional neural network
DSC: Dice similarity coefficient
HD: Hausdorff distance
IVC: Inferior vena cava
LHV: Left hepatic vein
MSD: Mean surface distance
MHV: Middle hepatic vein
PACS: Picture archiving and communication system
PVP: Portal venous phase
RHV: Right hepatic vein
RV: Volume ratio
S: Segment
VR: Volume rendering
2.5D: 2.5-Dimensional
